# Effect of foliar application of potassium on wheat tolerance to salt stress

**DOI:** 10.1371/journal.pone.0336407

**Published:** 2025-11-06

**Authors:** Fiza Noor, Humera Nawaz, Ameer Khan, Muhammad Yousaf Shani, Muhammad Azmat, Syed Mohsin Abbas, Iqra Arshad, Robina Aziz, Muhammad Saleem, Francesco De Mastro, Muhammad Yasin Ashraf, Gennaro Brunetti, Claudio Cocozza

**Affiliations:** 1 University of Education, Faisalabad Campus, Faisalabad, Pakistan; 2 Pakistan Institute of Engineering and Applied Sciences (PIEAS), Nuclear Institute for Agriculture and Biology College (NIAB-C), Islamabad, Pakistan; 3 Institute of Molecular Biology and Biotechnology (IMBB), The University of Lahore, Lahore, Pakistan; 4 Department of Horticulture, Faculty of Agricultural Sciences, University of Punjab Lahore, Lahore, Punjab, Pakistan; 5 Department of Botany, Government College Women University Sialkot, Sialkot, Pakistan; 6 Department of Soil, Plant, and Food Sciences, University of Bari “Aldo Moro”, Bari, Italy; Institute of Applied Sciences & Humanities, GLA University, INDIA

## Abstract

Salinity stress severely hampers wheat productivity by impairing growth, photosynthesis, and metabolic balance. Potassium nutrition, however, can mitigate these effects by supporting physiological and biochemical stability. This study assessed the impact of foliar potassium application (0, 200 and 400 ppm) on two wheat cultivars, Galaxy-13 and Uqab-2000, exposed to normal (0 mM NaCl) and saline conditions (100 and 150 mM NaCl, respectively). Salinity significantly reduced root and shoot growth, biomass, chlorophyll content, photosynthetic rate, and stomatal conductance. Potassium supplementation, particularly at 400 ppm, alleviated these reductions, with Galaxy-13 showing a 32.01% increase in shoot length and a 45.11% increase in shoot dry weight compared to Uqab-2000. Biochemical analyses revealed that Galaxy-13 sustained higher nitrate and nitrite reductase activities (6.23 and 3.63 μmol NO_2_ g^-1^ FW h^-1^, respectively) and total soluble proteins (10.1 mg g^-1^ FW), whereas Uqab-2000 accumulated more soluble sugars and free amino acids under stress (9.8 and 19.8 mg g^-1^ FW, respectively). Oxidative stress indicators (malondialdehyde and hydrogen peroxide) rose under salinity, but potassium reduced their levels, with Galaxy-13 exhibiting stronger antioxidant regulation. Nutrient profiling further demonstrated that Galaxy-13 maintained higher N, P, and K contents and minimized Na uptake, unlike Uqab-2000, which showed severe ionic imbalance. Multivariate analyses (PCA, heatmap, and correlation) highlighted strong positive associations of potassium, especially K400, with biomass accumulation, photosynthetic efficiency, and nutrient homeostasis. The findings establish that Galaxy-13 possesses superior salinity tolerance and responds more favorably to potassium nutrition. This study provides novel evidence that cultivar-specific potassium management can enhance wheat resilience in saline environments, offering a practical strategy for sustaining yield under stress.

## 1. Introduction

Wheat (*Triticum aestivum* L.) is one of the most widely cultivated cereals and a major dietary source for nearly 35% of the global population [1]. It is not only essential for global food and nutritional security but also holds significant economic importance, particularly in developing countries such as Pakistan. Here, wheat cultivation ensures livelihood security for about 65% of the population and contributes nearly 25% to the agricultural gross domestic product [2]. As a primary source of carbohydrates and dietary fiber, wheat plays a crucial role in human nutrition and metabolic functions [3]. Globally, it is grown on approximately 218 million hectares and serves as the staple crop for over one-fifth of the world’s population [4]. In Pakistan alone, wheat covers about 9.0 million hectares and represents the main source of food and income for 80% of farmers [5]. With the world’s population expected to reach 9.7 billion by 2050, enhancing wheat productivity has become an urgent priority for global food security [6].

Despite its significance, wheat productivity in many developing regions remains lower than the global average due to soil degradation and increasing abiotic stresses. Among these, soil salinity has emerged as a major threat, affecting nearly 25–30% of irrigated lands worldwide (954 million hectares) [7]. Salinity stress disrupts ion balance, photosynthesis, and water uptake, while promoting the accumulation of reactive oxygen species (ROS), which impair membrane stability, metabolic efficiency, and crop yields [8,9]. Although plants possess defence mechanisms, including antioxidant enzymes such as superoxide dismutase (SOD), peroxidase (POD), catalase (CAT), and ascorbate peroxidase (APX), as well as non-enzymatic osmolytes like proline and phenolics, these systems often fail to fully mitigate oxidative damage under severe stress [10].

Climate change is further aggravating soil salinization, especially in arid and semi-arid regions, thereby necessitating efficient management strategies to improve stress resilience [11,12]. Potassium (K⁺), one of the most essential plant macronutrients, plays a pivotal role in enhancing stress tolerance by maintaining ionic homeostasis, activating key enzymes, stabilizing membranes, and regulating stomatal conductance [13]. It is estimated that K⁺ activates more than 60 enzymes related to primary metabolism, growth regulation, and stress mitigation [14]. Under potassium-deficient conditions, protein synthesis and nitrogen assimilation are severely restricted due to ionic competition between Na⁺ and K⁺ at transport sites [15]. Compared with other osmolytes such as Na ⁺ , Cl ⁻ , proline, or glycine betaine, K ⁺ is more efficient in maintaining osmotic balance and sustaining enzymatic activities during stress [16].

Earlier studies have highlighted that combined stresses, such as salinity and nutrient deficiency, generate more severe impacts on plant metabolism and productivity than individual stressors [17]. Salinity significantly reduces K⁺ uptake because of the antagonistic interaction between Na⁺ and K ⁺ , resulting in impaired growth and yield [18]. Although various adaptive mechanisms in wheat have been explored, conventional practices have remained inadequate to mitigate this dual stress [19,20]. Recent evidence suggests that potassium fertilization can substantially improve crop resilience and productivity in salt-affected soils [21]. Foliar application of K ⁺ has gained attention for its efficiency in bypassing soil limitations and directly supplying nutrients to metabolic sites. Studies have reported that foliar potassium application enhances photosynthetic pigments, improves stomatal regulation, and maintains ionic balance under salinity stress [22–24].

To address these challenges, several strategies, including the development of salt-tolerant genotypes and improved nutrient management practices, have been proposed [25–27]. However, the interaction between salinity stress and K⁺ nutrition in wheat remains insufficiently understood, particularly under field-relevant conditions [28]. The present study was therefore designed to evaluate the ameliorative role of foliar-applied potassium in mitigating salinity stress in wheat. Specifically, it investigates how foliar K⁺ application influences osmolyte accumulation, chlorophyll retention, antioxidant activity, and nitrogen metabolism, while maintaining Na ⁺ /K⁺ homeostasis under saline and non-saline conditions. By comparing two wheat cultivars, this study provides critical insights into the combined effects of salinity and potassium nutrition. The findings are expected to contribute to the development of effective nutrient management strategies for sustaining wheat productivity in salt-degraded soils. The novelty of this work lies in its integrative assessment of physiological, biochemical, and nutrient partitioning responses under dual stress conditions, which has not been comprehensively addressed in earlier studies.

## 2. Materials and methods

### 2.1. Experimental setup

The trial was conducted in a greenhouse under controlled conditions at the Nuclear Institute for Agriculture and Biology (NIAB), Faisalabad, in collaboration with the Department of Botany, University of Education, Faisalabad Campus, during November 2021 to January 2022. A pot-culture was conducted in the NIAB greenhouse; to fill the pots, soil was collected from the NIAB Agriculture Farm field for the cultivation of crops. Prior to the commencement of the experiment, soil samples were collected from a 0–30 cm depth of the soil using an auger in a W-pattern to assess baseline soil conditions. Soil texture was determined using the method described by [29], and key physico-chemical properties, including pH, electrical conductivity (EC), organic carbon content, and nutrient levels, were analyzed following the procedures outlined by Jackson [30]. The results of these soil analyses are summarized in Table 1. Throughout the experimental period, the air temperature was 25 ± 2 °C during the daytime and 18 ± 2 °C at night, with relative air humidity fluctuating between 65–70%. These controlled environmental conditions were maintained to ensure optimal growth for the wheat varieties. Seeds of the wheat varieties Galaxy-13 and Uqab-2000 were procured from the Ayub Agricultural Research Institute (AARI), Faisalabad, Pakistan. To ensure uniformity, visually healthy seeds of consistent size and weight were selected. The experiment was conducted in plastic pots, each containing 6 kg of homogenized soil. Seeds of both wheat varieties were sown separately in each pot and allowed to grow for 20 days under controlled conditions. After successful germination, thinning was carried out to maintain five healthy seedlings per pot to ensure consistent growth conditions and avoid overcrowding. These seedlings were allowed to grow until they reached the appropriate growth stage for further treatment applications.

**Table 1:** Measurement of soil-related attributes used in the current investigation.

Soil Characteristics	Values
Saturation Percentage	40.6
pH_H2O_	7.84
Electrical Conductivity (dS m^ − 1^)	1.47
Organic C content (%)	0.74
Sodium (mg kg^ − 1^)	68
Chloride (meq L^ − 1^)	1.54
Field Capacity (mL)	406
Carbonate	Nil
Bicarbonate (meq L^ − 1^)	2.48
Soil Texture	Silt Loam
Magnesium (meq L^ − 1^)	4
Available Potassium (mg kg^ − 1^)	17
Calcium (meq L^ − 1^)	21

After the successful germination of wheat seedlings, the plants were subjected to salt stress treatments using three different solutions of NaCl, analytical grade (Merck, Germany): 0 mM, (soil EC = 1.47 dS m^−1^), 100 mM (moderate salinity stress, soil EC = 10.9 dS m^−1^), and 150 mM (severe salinity stress, soil EC = 15.8 dS m^−1^). These salt treatments were applied to simulate varying degrees of soil salinity stress, reflecting conditions that wheat plants may encounter in saline-affected agricultural environments. In addition to the salinity treatments, foliar potassium was applied at three concentrations: 0 ppm, 200 ppm, or 400 ppm (Table 2). Potassium foliar treatments were applied three times: initially when the seedlings were two weeks old, followed by two applications at tillering and booting stages. Each treatment consisted of applying 250 mL per pot of the corresponding K₂SO₄ solution to the foliage, ensuring uniform coverage of the plant’s leaves. Following the foliar supplementation, key physio-morphological and biochemical attributes were quantified using established protocols in wheat genotypes grown under both normal and stress conditions.

**Table 2:** Treatment combinations applied to evaluate the effect of potassium foliar application on wheat under salinity stress.

Treatments code	Description
T_1_	Control
T_2_	C + K 200 ppm
T_3_	C + K 400 ppm
T_4_	S100 mM
T_5_	S100mM + K 200 ppm
T_6_	S100mM + K 400 ppm
T_7_	S150 mM
T_8_	S150mM + K200 ppm
T_9_	S150mM + K400 ppm

### 2.2. Growth parameters

Several key growth parameters were assessed to evaluate the impact of salinity stress and potassium application on wheat varieties. These included shoot and root length, as well as the fresh and dry weight of both shoot and root samples. Root and shoot lengths were measured in centimeters using a calibrated ruler. These growth parameters were measured at regular intervals to monitor the effects of salinity stress and potassium treatments over time, and the data collected were used to calculate growth indices such as root-to-shoot ratio, relative growth rate, and stress tolerance index.

Fresh weight of the root and shoot samples was determined using a digital analytical balance (Model FA2104B, Sartorius). Subsequently, the root and shoot samples were oven-dried at a consistent temperature of 72°C for 72 hours in a drying oven (Model YPO-072, Yamato Scientific) until a constant weight was achieved, indicating complete dehydration. The dry weights of the root and shoot samples were then recorded using the same balance.

### 2.3. Physiological parameters

Physiological parameters were measured using an Infrared Gas Analyzer (IRGA, Model Ci-340, CID Bio-Science) to capture key photosynthetic processes. Measurements of net photosynthesis rate (Pn), stomatal conductance (SC), and transpiration rate (E) were recorded on fully exposed younger leaves under natural sunlight during peak daylight hours, specifically between 10:30 am and 01:00 pm. These time intervals ensured consistent light conditions, as the midday period is typically associated with stable environmental factors for photosynthesis. The measurements were conducted under controlled conditions to minimize environmental variability, and three replicates per treatment were taken for each physiological parameter. These parameters were assessed to understand the impact of salinity stress and potassium supplementation on wheat’s photosynthetic efficiency and water relations.

### 2.4. Biochemical parameters

Superoxide dismutase (SOD) activity was determined by measuring the inhibition of nitroblue tetrazolium photoreduction at 560 nm [31]. Peroxidase (POD) activity was measured by monitoring guaiacol oxidation at 470 nm. Catalase (CAT) activity was quantified by assessing the decomposition of H₂O₂ at 240 nm, following the method of [32]. Ascorbate peroxidase (APX) activity was assessed by recording ascorbate oxidation at 290 nm according to the procedure of [33]. Total soluble protein (TSP) content was estimated using Bradford’s method, with absorbance taken at 595 nm [34]. Total Free Amino Acids (TFA) were estimated using ninhydrin reagent, where the amino acids form a purple complex upon heating, measured at 570 nm [35]. Total soluble sugars (TSS) were quantified using the anthrone method at 620 nm [36]. Malondialdehyde (MDA) was determined through the thiobarbituric acid-reactive substances assay at 532 nm [37]. Hydrogen peroxide (H₂O₂) concentration was measured using its reaction with potassium iodide at 390 nm [38]. Nitrate reductase activity (NRA) was determined using the procedure of Sym [39], while nitrite reductase activity (NiRA) was measured according to Ramarao et al. [40]. Chlorophyll a (Chl a), chlorophyll b (Chl b), and carotenoids (Car) were extracted in 80% acetone, and absorbance was recorded at 663 nm, 645 nm, and 470 nm, respectively, using the method of Arnon [41]. Total chlorophyll (T.chl) is the sum of Chl a and b. All assays were performed in triplicate, and absorbance readings were obtained using a spectrophotometer (N6000SPLUS, China). Results were expressed as relative changes compared with the control, providing a comparative assessment of the biochemical and physiological responses to the treatments.

### 2.5. Wheat leaves nutrient contents

For nutrient analysis, wheat leaf samples were first rinsed thoroughly with tap water to remove any surface dust, followed by a second wash with distilled water to ensure purity. The cleaned samples were oven-dried at 70 ± 2 °C to a constant weight and then finely ground. Total Nitrogen (N) content was determined on such samples using the Bremner method [42].

The finely ground wheat leaves were subjected to acid digestion following standard protocols [5]. Then, the total Phosphorus (P) content was measured spectrophotometrically using Jackson’s method [43], Sodium (Na⁺) and Potassium (K⁺) concentrations were determined using a flame photometer (Jenway PFP7, Leicestershire, UK). All measurements were performed in triplicate, and the results were expressed in mg per gram of dry weight (mg/g DW) for each nutrient. The nutrient content data were used to assess the plant’s nutritional status and its response to each treatment.

### 2.6. *S*tatistical analyses

The experiment was conducted under a completely randomized design with a factorial arrangement consisting of two wheat cultivars, three salinity levels (0, 100, and 150 mM NaCl), and three potassium foliar treatments (0, 200, and 400 ppm). Each combination of treatments was replicated three times. Statistical significance between the treatments and the wheat varieties (Galaxy-13 and Uqab-2000) was evaluated using a two-way analysis of variance (ANOVA), followed by Tukey’s Honest Significant Difference (HSD) test for pairwise comparisons. The analysis was conducted using Statistics 8.1 software. This approach allowed for the determination of significant differences in growth, physiological, and biochemical responses among the different treatment groups and varieties. Principal component analysis (PCA) was performed to assess the relationships among physio-morphological and biochemical traits under varying treatments. PCA was carried out using R software (version 4.3.1), utilizing the FactoMineR and factoextra packages. PCA provided insights into the positive and negative interactions between the wheat varieties (Galaxy-13 and Uqab-2000) and the imposed stress treatments. To better understand the contribution of each principal component (PC) to the total variability, scree plot analysis was conducted separately for each wheat cultivar. For an in-depth examination of genotype-specific responses to varying stress intensities, hierarchical clustering was performed using heatmap analysis in Pheatmap (R v4.4.2). This approach helped visualize the differential responses of the wheat varieties across the different stress regimes, highlighting genotype-specific patterns of adaptation. Additionally, the multi-trait genotype-ideotype distance index (MGIDI), implemented through the metan package in R, was used to quantitatively rank the genotypes (Galaxy-13 and Uqab-2000) for drought tolerance under each specific stress condition. The MGIDI provided a comprehensive evaluation of the overall performance of the wheat varieties in terms of their ability to tolerate drought stress, helping to identify superior genotypes under each treatment condition. All statistical analyses were performed at a significance level of p ≤ 0.05.

## 3. Results

### 3.1. Growth parameters

**Fig 1:**
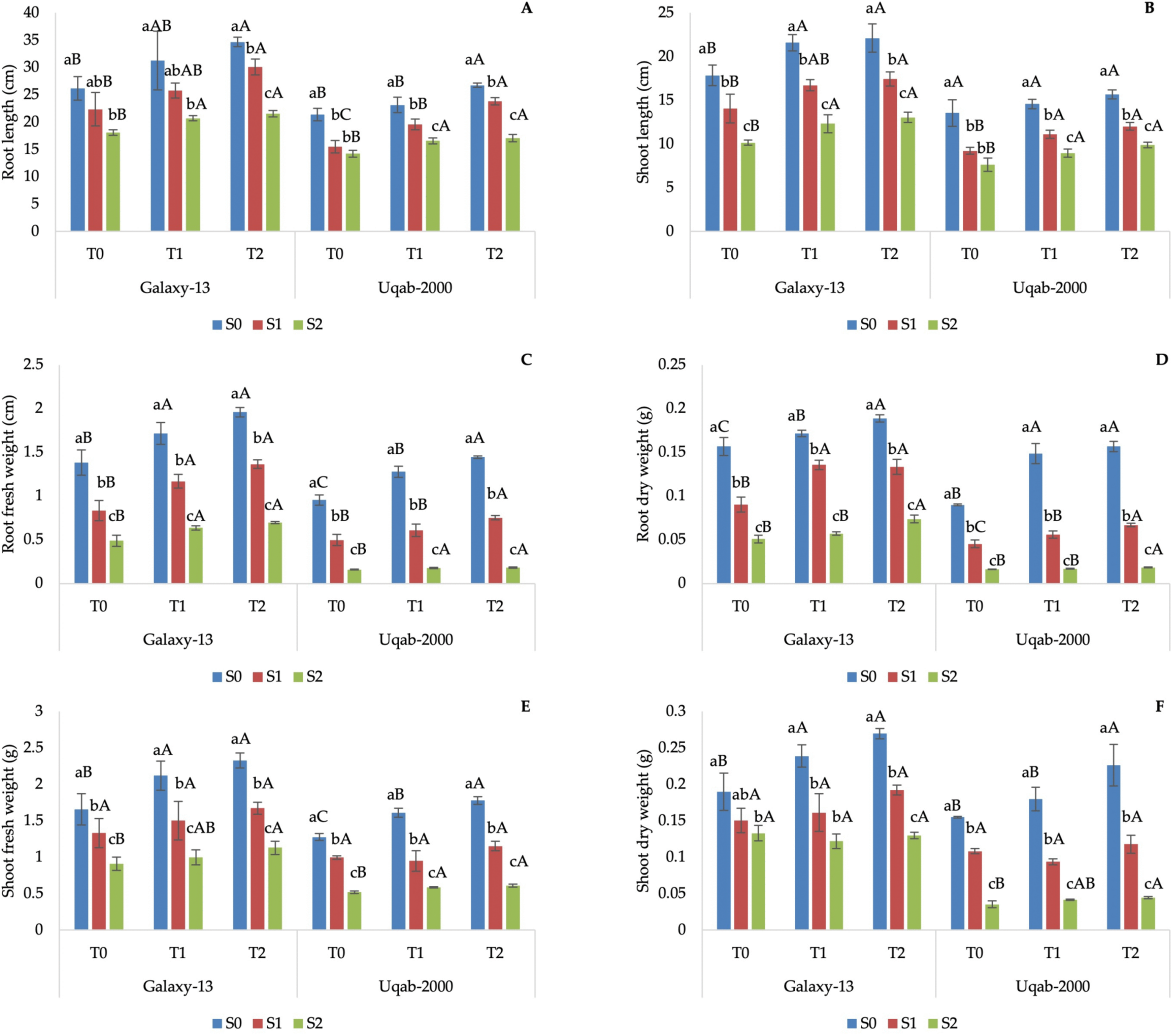
Impact of potassium nutrition on root length (A), shoot length (B), root fresh weight (C), root dry weight (D), shoot fresh weight (E), and shoot dry weight (F) in Galaxy-13 and Uqab-2000 wheat cultivars under normal and saline conditions. Lowercase letters represent differences between saline conditions (S_0_, S_1_, and S_2_) under the same foliar potassium application. Uppercase letters represent differences between foliar potassium application (T_0_, T_1_, and T_2_) under the same saline condition. Error bars represent the standard deviation of the mean for each measurement.

Growth parameters were significantly (P ≤ 0.05) affected by each treatment (Table 3), and Galaxy-13 performed better with the combination S_0_ + T_2_, followed by S_0_ + T_1_ and S_0_ + T_0_ (Fig 1). The Uqab-2000 variety showed lower growth parameters than the other, but with the same trends (Fig 1), meaning that the foliar application of potassium alleviated some of the negative effects of salinity.

**Table 3:** Mean sum of squares values of the physio-morphic and biochemical traits of Galaxy-13 and Uqab-2000 wheat cultivars subjected to different levels of salinity stress and potassium nutrition.

SOV	DF	SL	RL	RFW	RDW	SFW	SDW	Chl.a	Chl.b	T.chl	Car	SC	E	Pn	NRA	NiRA	TSP	TSS	TFA
V	1	302.93***	462.88***	2.94***	0.03***	2.89***	0.05***	0.48***	0.43***	1.84***	0.09***	0.26***	26.6***	245.3***	39.3***	47.5***	159.4***	32.6***	117.9***
S	2	236.7***	379.8***	5.12***	0.05***	4.51***	0.07***	0.9***	0.62***	3.03***	0.05***	0.39***	39.9***	412.6***	96.8***	57.9***	275.2***	62.8***	248.1***
T	2	41.88***	164.04***	0.55***	0.004***	0.49***	0.006***	0.06***	0.04***	0.21***	0.002ns	0.05***	4.9***	58.2***	13.53***	5.3***	40.05***	7.6***	20.2***
V × S	2	10.48***	9.87ns	0.004ns	0.0006***	0.001ns	0.002**	0.01***	0.009***	0.016**	0.003ns	0.002**	0.17*	2.5**	1.36**	1.9**	2.76***	2.8***	5.3***
V × T	2	2.6^ns^	1.56ns	0.03**	0.00002ns	0.03ns	0.0001ns	0.003**	0.003**	0.012**	0.0006ns	0.007***	0.26**	3.5***	0.85*	0.18ns	2.36***	0.01ns	0.2ns
S × T	4	0.23ns	10.67**	0.06***	0.0007***	0.09**	0.002***	0.008***	0.009***	0.03***	0.002ns	0.005***	0.23**	1.19**	1.56**	1.66**	0.708**	2.47***	9.1***
V × S × T	4	0.49ns	2.03ns	0.005ns	0.0006***	0.003ns	0.0003ns	0.005**	0.005**	0.02**	0.0005ns	0.0003**	0.17*	0.61*	1.72**	1.21**	0.84**	0.29**	1.14**
Error	36	0.79	3.08	0.004	0.00003	0.01	0.0002	0.0007	0.0007	0.002	0.001	0.0005	0.04	0.24	0.25	7.29	0.121	0.05	0.218
Total	53																		

Significant differences among the salinity stress and potassium nutrition treatments: p < 0.001 ***, p < 0.01 **, p < 0.05 *, and ns (non-significant); V, variety; S, stress; T, treatments; V × S, interaction between variety and stress; V × T, interaction between variety and treatment; V × S × T, interaction among varieties, stress and, treatments; DF, degree of freedom; and SOV, significance of variance. SL, shoot length; RL, root length; RFW, root fresh weight; RDW, root dry weight; SFW, shoot fresh weight; SDW, shoot dry weight; Chl.a, chlorophyll a; Chl.b, chlorophyll b; T.chl, total chlorophyll; Car, carotenoid contents, SC, stomatal conductance; E, transpiration rate; Pn, photosynthesis rate; NRA, nitrate reductase activity; NiRA, nitrite reductase activity; TSP, total soluble proteins; TSS, total soluble sugars; and TFA, total free amino acid.

### 3.2. Physio-biochemical attributes

**Fig 2:**
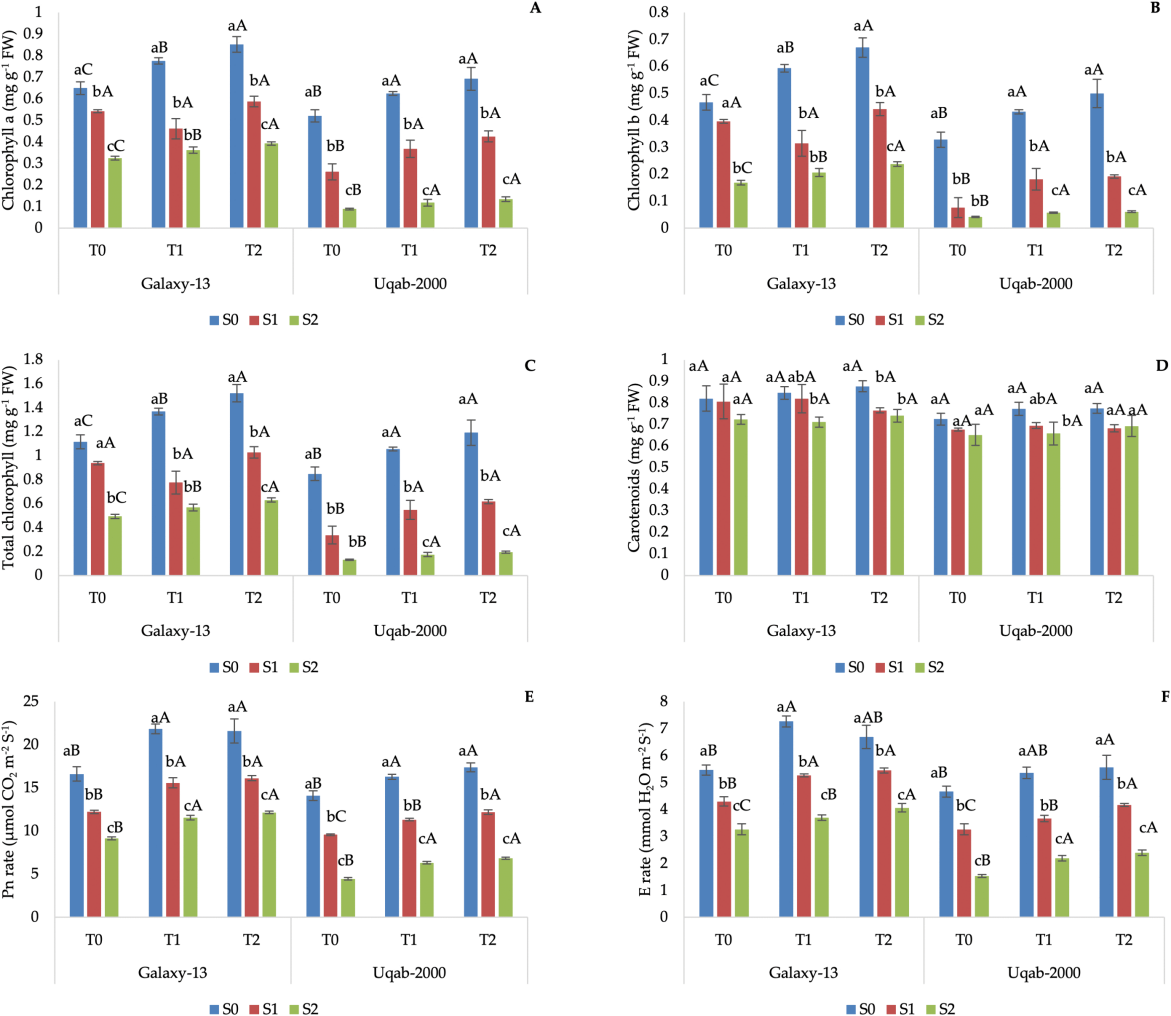
Impact of potassium nutrition on chlorophyll a content (A), chlorophyll b content (B), total chlorophyll content (C), carotenoids content (D), photosynthesis rate (E), and transpiration rate (F) in Galaxy-13 and Uqab-2000 wheat cultivars under normal and saline conditions. Lowercase letters represent differences between saline conditions (S_0_, S_1_, and S_2_) under the same foliar potassium application. Uppercase letters represent differences between foliar potassium application (T_0_, T_1_, and T_2_) under the same saline condition. Error bars represent the standard deviation of the mean for each measurement.

Galaxy-13 showed the highest content of all chlorophylls with the treatment S0 + T2 (Fig 2A,B,C), while its Pn reached the highest values with S0 + T1 and S0 + T2 treatments, without any significant differences between the two K applications (Fig 2E). The highest E was reached with the combination S_0_ + T_1_ (Fig 2F), while the highest Car content was obtained without salt application (S0), regardless of the K application (Fig 2D). In general, saline stress decreased the above parameters, although the chlorophyll content did not change significantly within the S1 treatments, and the highest application of K better alleviated the stress induced by the highest salinity (S2; Fig 2A,B,C). Pn and E were the same with the lowest K application, regardless of the levels of saline stress (S1 or S2; Fig 2E,F). Finally, the Car content was the same with both saline levels of stress, regardless of the quantity of K applied (Fig 2D).

Uqab-2000 resulted in lower physio-biochemical parameters than Galaxy-13. The highest chlorophylls content, Pn and E were obtained without saline stress and with the K application, regardless of its quantity (Fig 2A,B,C,E,F). The Car content resembled the trends observed for Galaxy-13 treated with T_0_ and T_1_, regardless of the saline stress (Fig 2D). The saline stress impacted negatively on the above parameters even for the Uqab-2000 variety. The T.chl content reduced when the saline stress increased but did not change between the two K applications (Fig 2A,B,C). Pn and E ameliorated with the highest K application when subjected to S1 stress, while they did not show significant differences when stressed with the highest salinity (S2), regardless of the K quantity applied (Fig 2E,F).

**Fig 3:**
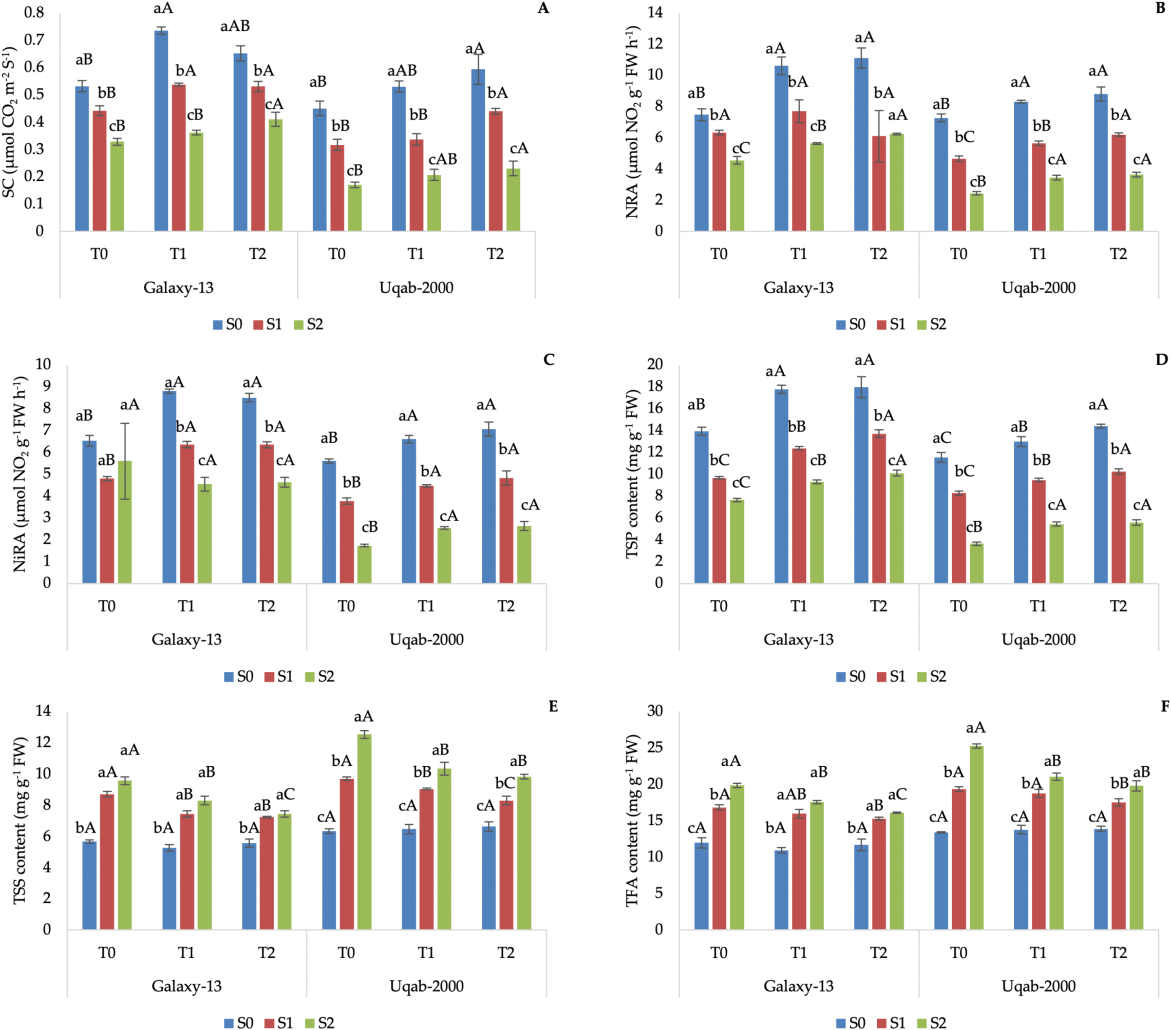
Impact of potassium nutrition on stomatal conductance (A), nitrate reductase activity (B), nitrite reductase activity (C), total soluble proteins (D), total soluble sugars (E), and total free amino acids (F) in Galaxy-13 and Uqab-2000 wheat cultivars under normal and saline conditions. Lowercase letters represent differences between saline conditions (S_0_, S_1_, and S_2_) under the same foliar potassium application. Uppercase letters represent differences between foliar potassium application (T_0_, T_1_, and T_2_) under the same saline condition. Error bars represent the standard deviation of the mean for each measurement.

In general, Galaxy-13 showed the highest values of SC, NRA, NiRA, and TSP regardless of treatment (Fig 3A,B,C and D). In contrast, TSS and TFA were higher without K and under the maximum saline stress (S_2_; Fig 3E,F). With saline irrigations, SC, NRA, NiRA, and STP decreased in proportion to the increase in salt concentration, while TSS and TFA showed behaviours inversely proportional to irrigation water salinity (Fig 3). Ubaq-2000 showed lower values of SC, NRA, NiRA, TSP, TSS and TFA than Galaxy-13 cultivar, and the two varieties shared the same trends of each parameter (Fig 3).

### 3.3. Markers of oxidative stress

**Fig 4:**
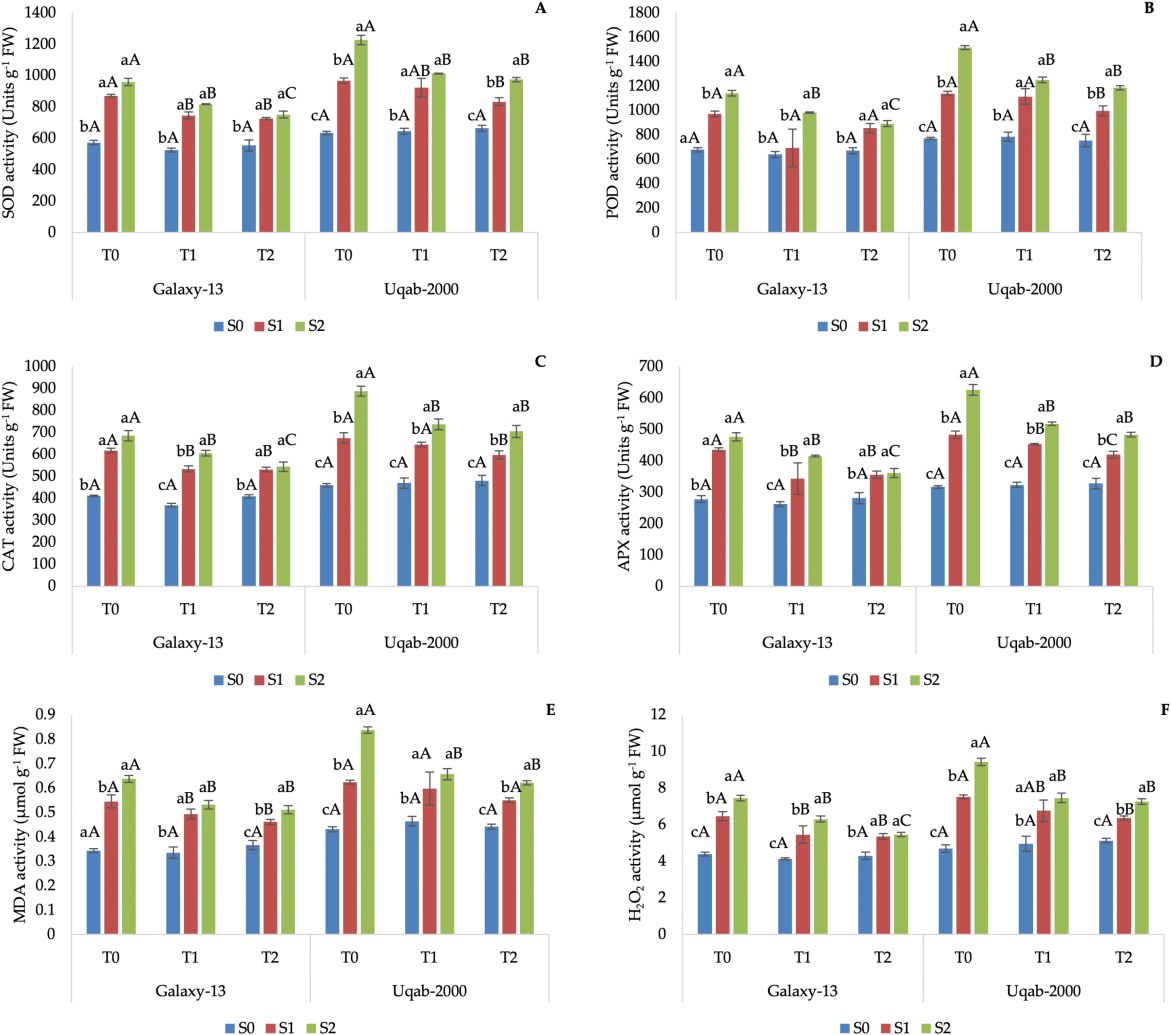
Impact of potassium nutrition on superoxide dismutase activity (A), peroxidase activity (B), catalase activity (C), ascorbate peroxidase activity (D), malondialdehyde content (E), and hydrogen peroxide activity (F) in Galaxy-13 and Uqab-2000 wheat cultivars under normal and saline conditions. Lowercase letters represent differences between saline conditions (S_0_, S_1_, and S_2_) under the same foliar potassium application. Uppercase letters represent differences between foliar potassium application (T_0_, T_1_, and T_2_) under the same saline condition. Error bars represent the standard deviation of the mean for each measurement.

Oxidative stress markers (MDA, H₂O₂) and antioxidant enzyme activities (SOD, POD, CAT, APX) increased sharply under high salinity, particularly without potassium (Table 4). Potassium supplementation reduced oxidative damage in both cultivars, with Galaxy-13 showing lower oxidative stress than Uqab-2000 (Fig 4).

**Table 4:** Mean sum of squares values of enzymatic activities and nutrient contents of Galaxy-13 and Uqab-2000 wheat cultivars subjected to different levels of salinity stress and potassium nutrition.

SOV	DF	SOD	POD	CAT	APX	MDA	H202	N	P	K	Na/K
V	1	308100***	657587***	151580***	91761***	0.16***	17.5***	0.21***	0.106ns	88.1***	2.5***
S	2	600878***	897321***	313725***	152607***	0.25***	32.1***	0.37***	1.28***	475.1***	30.9***
T	2	72712***	110399***	30941***	206559***	0.02***	5.1***	0.04***	0.32**	1188.7***	22.1***
V × S	2	21590***	48938***	11889***	6765***	0.004**	1.09***	0.002*	1.07***	4.52***	0.6***
V × T	2	614ns	12748**	260ns	261ns	0.001*	0.01ns	0.003**	0.16*	20.27***	0.2***
S × T	4	22663***	32487***	11122***	7202***	0.015^ns^	2.007***	0.002**	0.23**	17.9***	5.1***
V × S × T	4	2858**	14492**	1914**	1152*	0.001**	0.21**	0.002**	0.14**	3.8***	0.09***
Error	36	537	2176	322	255	0.0004	0.063	0.0005	0.04	0.31	0.008
Total	53										

SOD, superoxide dismutase activity; POD, peroxidase activity; CAT, catalase activity; APX, ascorbate peroxidase activity; MDA, malondialdehyde content; H_2_O_2_, hydrogen peroxide activity; N, nitrogen concentration; P, phosphorus concentration; K, potassium concentration; and Na/K, sodium potassium ratio.

### 3.4. Leaves nutrient content

**Fig 5:**
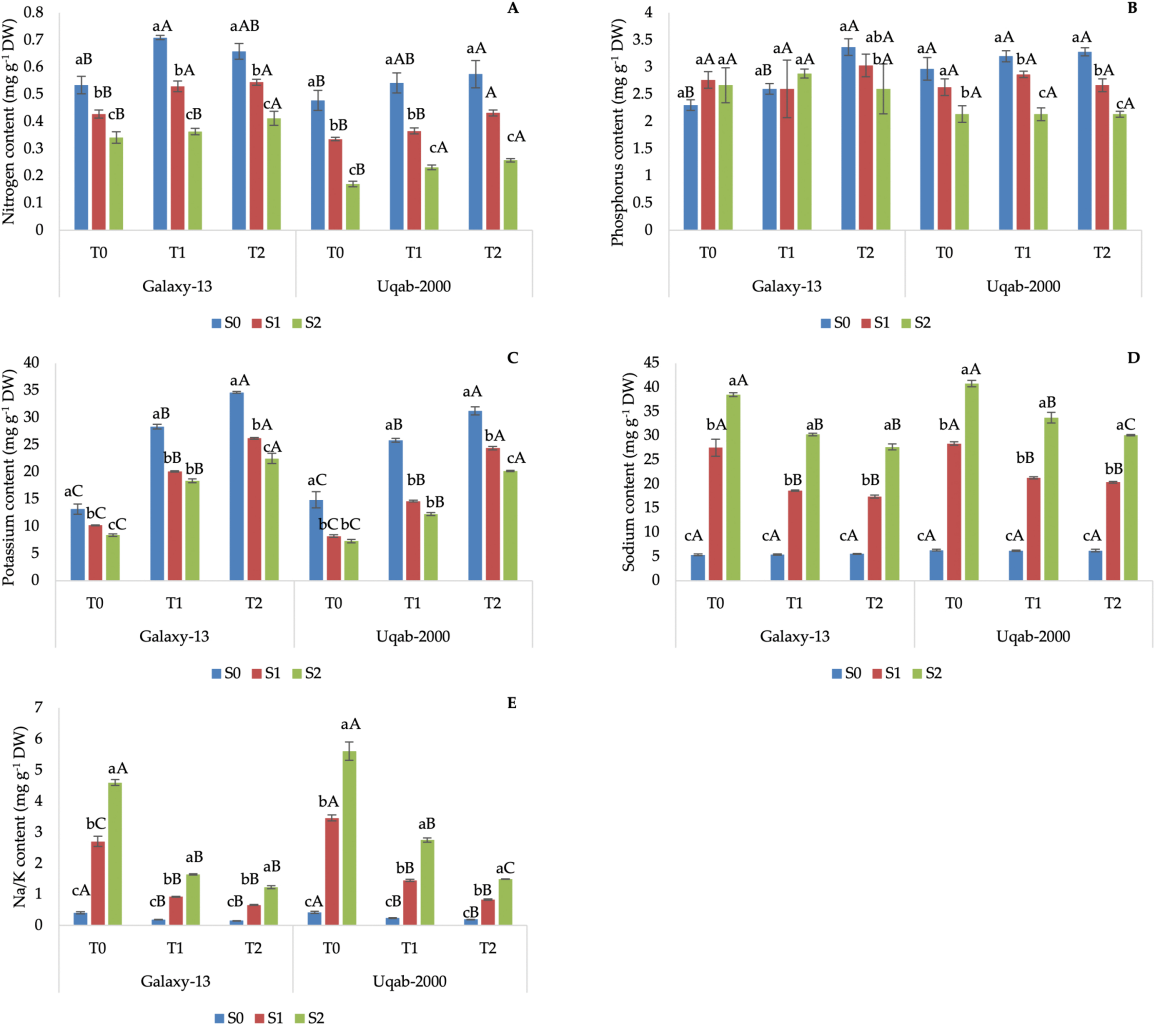
Impact of potassium nutrition on nitrogen contents (A), phosphorus contents (B), potassium contents (C), sodium contents (D), and sodium potassium ratio (E) in Galaxy-13 and Uqab-2000 wheat cultivars under normal and saline conditions. Lowercase letters represent differences between saline conditions (S_0_, S_1_, and S_2_) under the same foliar potassium application. Uppercase letters represent differences between foliar potassium application (T_0_, T_1_, and T_2_) under the same saline condition. Error bars represent the standard deviation of the mean for each measurement.

Galaxy-13 showed the highest N content under non-saline irrigation with K supplementation, while salinity proportionally decreased leaf N (Fig 5A). K content increased with foliar application under non-saline conditions but declined with saline stress (Fig 5C). The Na concentration was the lowest when Galaxy-13 was irrigated with non-saline water, regardless of the K supplementation, reached its maximum with the combination S2 + T0, but reduced with S2 irrigation and K supplementation (Fig 5D). P content showed little variation across treatments (Fig 5B). Ubaq-2000 displayed a similar pattern to Galaxy-13, but with lower N and K and comparable Na.

### 3.5. Principal component analysis

**Fig 6:**
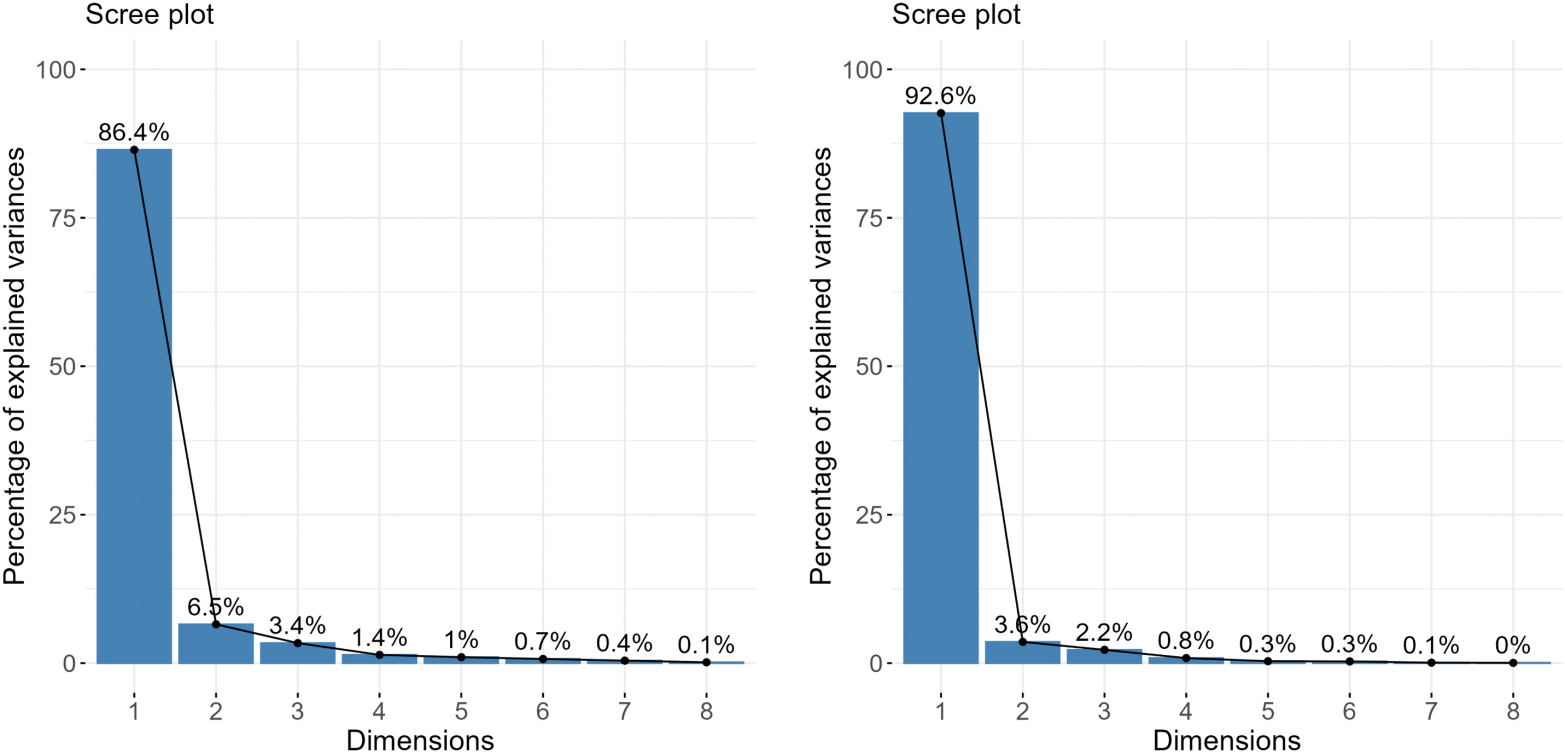
A scree plot analysis underscoring distinct factors on the x-axis, and eigenvalues on the y-axis for all the principal components (PCs) of studied wheat varieties: (A) Galaxy-13 and (B) Uqab-2000 grown under normal and saline environments.

**Fig 7:**
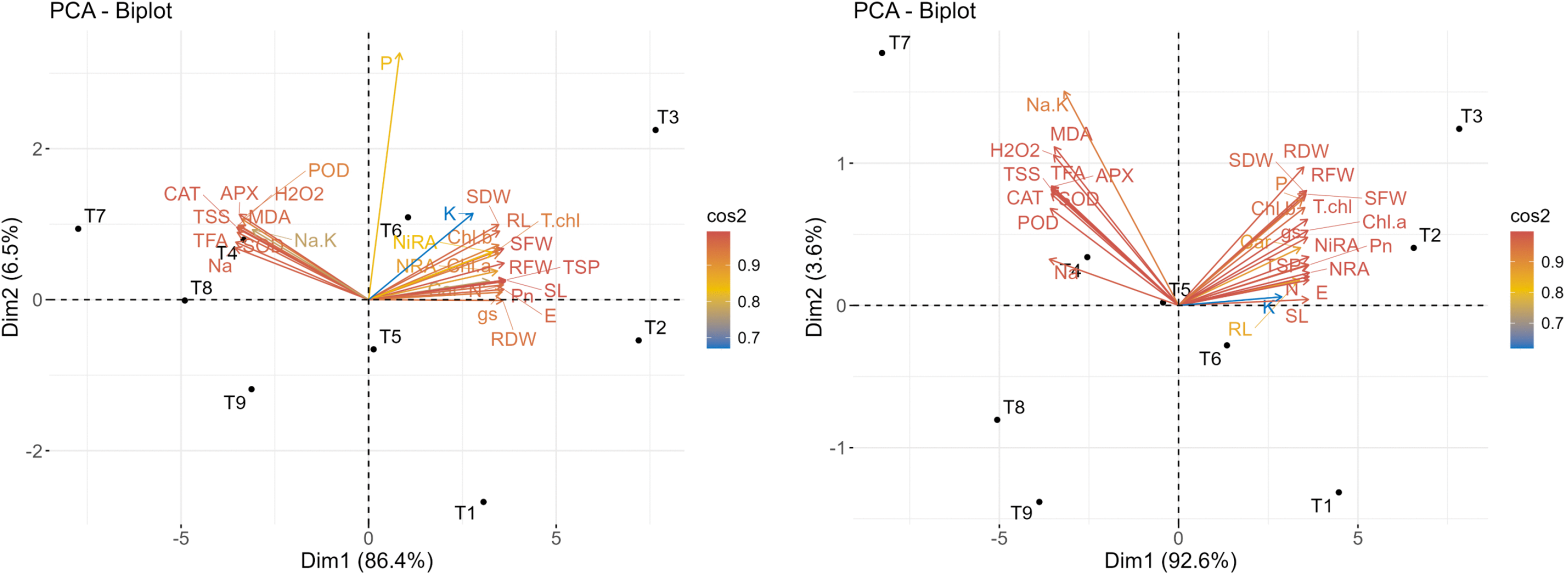
Principal component analysis (Biplot) for studied wheat varieties: (A) Galaxy-13 and(B) Uqab-2000, grown under normal and saline conditions.

Principal component analysis (PCA) was performed separately for both wheat cultivars to assess their responses under different salinity levels and potassium supplementation (Fig 6, 7). For Galaxy-13, the scree plot indicated that PC1 and PC2 explained 86.4% and 6.5% of the total variation, respectively (Fig 6A). The biplot showed a strong positive association of T3 (C + K400) with growth traits such as shoot and root length, fresh and dry weights, and shoot fresh weight. T6 (S100 mM + K400) was linked with higher phosphorus and potassium contents, while T7 (S150 mM) was associated with enhanced antioxidant enzyme activities (SOD, POD, CAT, APX), MDA, H₂O₂, and sodium concentration. A negative relationship was observed between growth and pigment traits (SL, RL, SFW, SDW, RFW, RDW, Chl.a, Chl.b, TSP, NRA, NiRA) and stress-related enzymatic attributes, as indicated by vectors in opposite directions (Fig 7A).

For Uqab-2000, PC1 and PC2 accounted for 92.6% and 3.6% of the variation, respectively (Fig 6B). The biplot showed that T2 (C + K200) and T3 (C + K400) were strongly associated with growth parameters (SL, RL, SFW, SDW, RFW, RDW, Pn, gs, E) and biochemical traits (TSP, total chlorophyll, carotenoids, NRA, NiRA). Conversely, T4 (S100 mM), T5 (S100 mM + K200), and T7 (S150 mM) clustered with antioxidant enzyme activities (SOD, POD, CAT, APX), MDA, H₂O₂, and sodium traits (Na and Na/K ratio), showing an inverse relationship with growth and pigment parameters (Fig 7B).

### 3.6. Heatmap analysis

**Fig 8:**
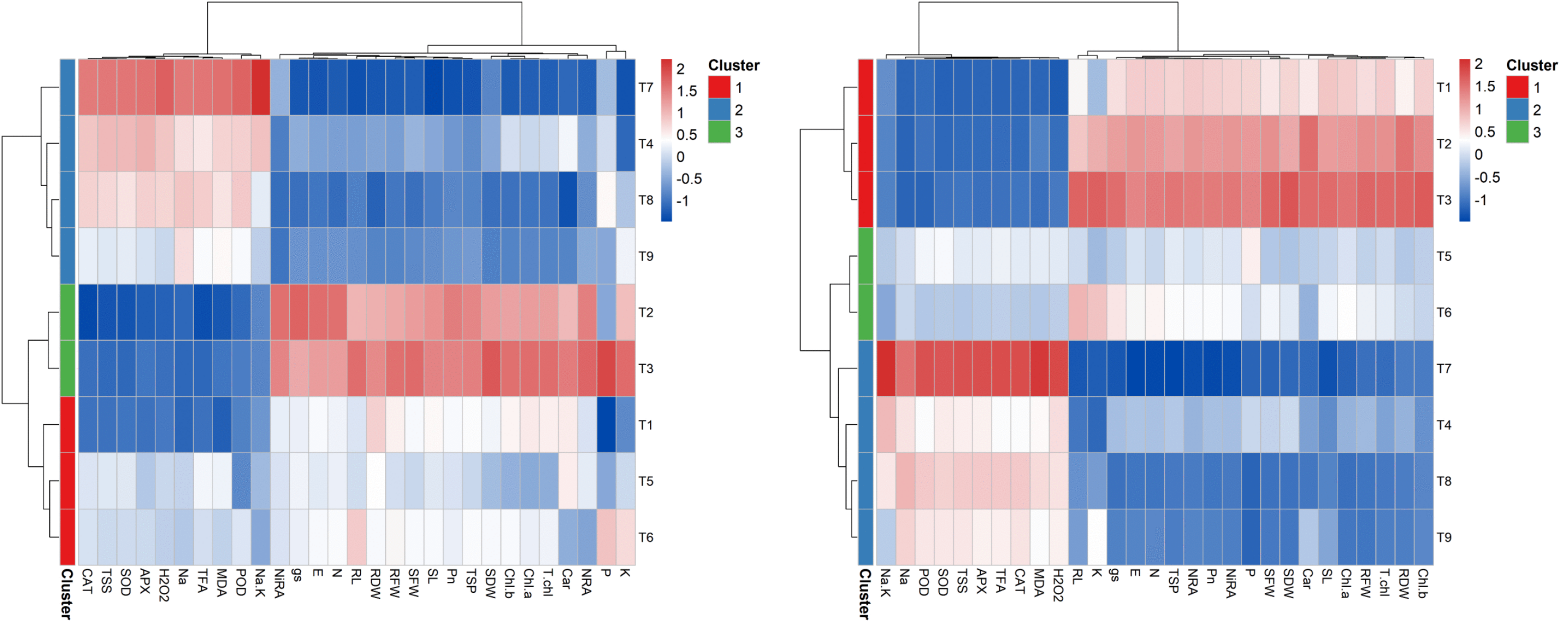
Heatmap analysis depicted clustering among imposed treatments and their interaction with analyzed traits in both wheat cultivars (A) Galaxy-13, (B) Uqab-2000.

Heatmap analysis was performed separately for both wheat cultivars to assess the associations among traits and treatments (Fig 8). For Galaxy-13, three major clusters were identified. The first cluster (T1, T5, T6) showed negative associations with chlorophylls, TSS, TSP, and TFA, but positive links with root length, phosphorus, and potassium. Within this cluster, T1 showed a strong negative correlation with TSS, antioxidant enzymes (CAT, APX), MDA, and mineral contents. The second cluster (T2, T3) was strongly associated with growth traits (SL, RL, SFW, SDW, RFW, RDW), gas exchange parameters (Pn, SC, E), and pigments, but negatively correlated with antioxidant enzymes, TSS, TSP, Na, H₂O₂, and MDA. The third cluster (T4, T7, T8, T9) showed strong positive associations with antioxidant enzymes, TSS, and H₂O₂, but negative associations with growth attributes.

In Uqab-2000, three distinct clusters were also observed. The first cluster (T1, T2, T3) was positively associated with growth, photosynthesis-related traits, pigments, and protein content, while negatively related to antioxidant enzymes and ROS. The second cluster (T5, T6) showed generally weak associations, except for a slight positive link with root length and potassium. The third cluster (T4, T7, T8, T9) displayed strong positive correlations with antioxidant enzymes, ROS, and sodium, but negative associations with morphological and pigment traits. Notably, T7 (S150 mM) showed the strongest negative relationship with growth and pigments, while being positively linked with oxidative stress traits.

### 3.7. Pearson’s correlation analysis

**Fig 9:**
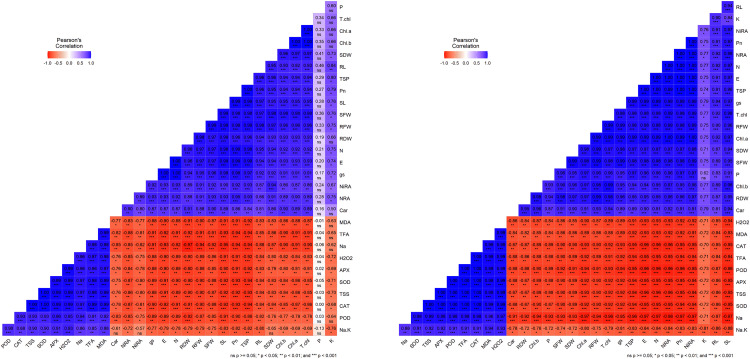
Pearson’s correlation analysis revealed positive and negative interplay among imposed treatments and their interaction with analyzed traits in both wheat cultivars (A) Galaxy-13, (B) Uqab-2000.

Pearson’s correlation analysis highlighted positive and negative relationships among traits in both wheat cultivars (Fig 9). For Galaxy-13, strong positive correlations were observed among growth traits and pigments. RFW was strongly correlated with Chl.a, Chl.b, SDW, RL, and TSP. Similarly, Pn was positively associated with Chl.b, SDW, and RL. In contrast, SOD showed negative correlations with SFW, RFW, E, and Car. Na correlated positively with MDA but negatively with RDW, N, E, and NiRA.

For Uqab-2000, SC was strongly correlated with shoot and root length, potassium, NiRA, N, and E. TFA were negatively associated with RFW, Chl.a, SFW, and phosphorus, but positively correlated with H₂O₂ and MDA. Sodium ions showed strong negative correlations with growth traits, pigments, and protein-related attributes, while displaying positive associations with oxidative stress indicators (H₂O₂, MDA, CAT, TSS, SOD).

## 4. Discussion

### 4.1. Effect of salinity on wheat parameters

This investigation provides compelling evidence of the ameliorative effects of foliar potassium application on wheat subjected to salinity stress. Salinity markedly reduced root and shoot lengths, as well as fresh and dry biomass, confirming earlier reports that highlight salinity as a major constraint to plant development [44,45]. Excess sodium ions (Na⁺) disturb the ionic equilibrium within plant cells, negatively affecting osmotic adjustment and protein synthesis, leading to reduced biomass [46]. These results are in line with the observations of [47], who emphasized the adverse impact of salinity on plant metabolism and productivity. Moreover, the accumulation of reactive oxygen species (ROS) under salt stress (Fig 4E-F) contributes to oxidative damage in cellular components, exacerbating growth reduction, a phenomenon corroborated by earlier studies [29,48,49].

### 4.2 Effect of potassium fertilization on wheat parameters

This study provides strong evidence that foliar potassium application significantly enhances wheat performance under salinity stress, with important implications for improving productivity in salt-affected regions. Both 200 ppm and 400 ppm concentrations significantly improved key growth parameters, even under severe salinity. This aligns with potassium’s known roles in osmotic regulation and ion transport, which are crucial for maintaining cellular function during stress 30 [26]. By fostering a favorable Na ⁺ /K⁺ ratio in the cytosol, potassium minimizes sodium toxicity and supports efficient nutrient assimilation, enhancing overall plant performance [19,50]. The present findings are consistent with those of [51,52], who reported improved plant growth in saline conditions following potassium supplementation, attributed to the stabilization of physiological and metabolic processes. Similarly, [53] demonstrated that potassium applied through seed soaking and foliar spraying significantly improved overall soybean growth, photosynthetic efficiency, K⁺ content, the K ⁺ /Na⁺ ratio, and enzymatic antioxidant activity, as well as enhanced yield and quality traits of soybean. Our results also extend current knowledge by showing that elevated potassium (400 ppm) was especially effective for Galaxy-13 variety under severe salinity. These cultivar-specific differences suggest genetic variation underlying potassium uptake, compartmentalization, and transporter efficiency, mechanisms that have been less emphasized in previous research. Similar studies have reported that higher potassium concentrations (500 ppm) positively affect the growth of herbaceous plants such as sunflower, and markedly enhance stomatal conductance, transpiration rate, water-use efficiency, CO₂ assimilation rate, total soluble proteins, and chlorophyll pigments [54]. Furthermore, our results underscore potassium’s ability to activate defense enzymes (SOD, POD, CAT, APX) and stabilize chlorophyll, thereby protecting photosynthetic machinery [55–58]. These benefits reflect potassium’s contribution to membrane stability, antioxidant defense, osmotic adjustment, and protein synthesis and overall plant health, as similarly reported by [59]. These protective effects are consistent with previous findings, but the present study also demonstrates that the lowest dose of potassium (200 ppm) has similar efficacy in the presence of high concentrations of NaCl. The beneficial impact of elevated potassium levels on plant vigor and physiological traits under salt stress concurs with the observations of [53], who reported reduced salinity-induced damage through potassium-driven improvement in growth and metabolism. Statistical analyses confirmed that exogenous potassium application significantly mitigated salt-induced damage by preserving membrane integrity, optimizing ion homeostasis, and supporting antioxidant defense mechanisms. Heatmap visualization emphasized T2 (control + 200 ppm K) and T3 (control + 400 ppm K) as the most favorable treatments, with clear enhancements in physiological and biochemical attributes. Pearson’s correlation analysis highlighted both synergistic and antagonistic interactions among traits. For example, peroxidase (APX) activity exhibited a negative correlation with growth traits, illustrating the metabolic burden associated with stress tolerance and the potential of potassium to ease this burden through oxidative stress mitigation. In short, potassium foliar application, especially at 400 ppm, proved highly beneficial in enhancing growth, protecting against oxidative injury, and maintaining physiological stability under salinity. These findings support the integration of potassium-based interventions into salinity management strategies for wheat production.

### 4.3. Current limitations and future perspectives

Despite these promising outcomes, some limitations should be acknowledged. First, the study was conducted under controlled conditions, which may not fully capture the variability of field environments where salinity interacts with other stresses. Second, only two wheat cultivars were examined, restricting the generalization of results across diverse germplasm. Finally, molecular insights, such as the expression of potassium and sodium transporters or signaling pathways, were not included but could help explain the observed cultivar-specific differences. Future research should therefore focus on multi-location field trials involving a wider range of wheat genotypes, coupled with molecular and omics-based approaches, to identify the genetic basis of potassium-mediated salinity tolerance. Such integrated studies will advance the development of salt-resilient wheat and guide nutrient management practices for sustainable crop production in salt-affected soils. This study’s novelty lies in its detailed evaluation of potassium’s dose-dependent and cultivar-specific effects under saline stress, supported by multivariate analyses. The findings highlight potassium foliar sprays as a practical and scalable solution to mitigate salinity-induced damage, contributing to sustainable agriculture and food security in regions increasingly affected by soil salinization.

## 5. Conclusions

This study demonstrates that foliar application of potassium at specific phenological stages of wheat is an effective strategy to mitigate the adverse effects of salinity stress. Potassium supplementation improved growth, biomass accumulation, antioxidant defense, and ion homeostasis, thereby enhancing overall stress tolerance. Among the tested cultivars, Galaxy-13 exhibited superior performance, particularly under higher potassium doses, indicating its potential as a salt-tolerant genotype for saline environments. These findings highlight the importance of cultivar-responsive nutrient management as a practical approach for sustaining wheat productivity in salt-affected soils. Future research should focus on multi-season and field-level validation, assessing economic feasibility, and evaluating grain yield and quality to strengthen the applicability of these findings for large-scale agricultural systems.

## Supporting information

S1 Table Experiment data set. (XLSX)
